# Diagnostic performance of abbreviated MRI in surveillance of patients with hepatocellular carcinoma candidates for liver transplantation

**DOI:** 10.1007/s11547-026-02259-0

**Published:** 2026-07-13

**Authors:** Monica Mattone, Alessandro De Maio, Alessandro Napoli, Carlo Catalano

**Affiliations:** Department of Radiological Sciences, Oncology and Pathology, Policlinico Umberto I, Sapienza University, Rome, Italy

**Keywords:** HCC, AMRI, HBP-AMRI, Dyn-AMRI, Liver transplantation

## Abstract

**Purpose:**

To compare the diagnostic performance of three different abbreviated MRI protocols simulated by extraction from a standard liver MRI protocol with hepatobiliary-specific contrast agent obtained for hepatocellular carcinoma (HCC) surveillance in patients candidates for liver transplantation.

**Methods:**

We conducted a retrospective study on cirrhotic patient population, undergoing MRI surveillance for HCC. All patients were candidates to liver transplantation with a total of 105/195 patients previously treated with liver-directed therapy. From the complete liver MRI with hepatobiliary-specific contrast agent (cMRI), three simulated abbreviated MRI protocols were extracted: dynamic contrast-enhanced abbreviated MRI (Dyn-AMRI); hepatobiliary phase abbreviated MRI (HBP-AMRI) and non-enhanced abbreviated MRI (NE-AMRI). Three abdominal radiologists independently interpreted the simulated protocols.

**Results:**

195 cirrhotic patients, candidates for liver transplantation, were analyzed. The overall sensitivity, specificity and accuracy of AMRI for HCC detection were 94%, 96% and 94% for Dyn-AMRI, 93%, 94% and 93% for HBP-AMRI, 90%, 90% and 90% for NE-AMRI. The interchangeability analysis revealed no significant difference between HBP-AMRI and cMRI and between cMRI and Dyn-AMRI. Regarding equivalence, agreement and interchangeability analysis, no significant differences were found in detection of HCC in patients previously undergoing liver-directed therapy.

**Conclusion:**

Both Dyn-AMRI and HBP-AMRI could be used in surveillance of cirrhotic patients, even after previous liver-direct therapy.

## Introduction

Liver transplantation represents the optimal curative treatment for hepatocellular carcinoma (HCC) arising in cirrhotic patients, offering a 5-year survival rate of 60–70%. However, the number of patients requiring liver transplantation far exceeds the availability of donors, making careful patient selection essential. Established criteria, including Milan, UCSF (University of California, San Francisco) and Toronto criteria [1–3], guide selection based on tumor size and number, while bridging therapies such as transarterial chemoembolization (TACE), radiofrequency ablation (RFA), microwave ablation (MWA) and stereotactic body radiation therapy (SBRT) are routinely employed to prevent tumor progression during the waiting period.

In this context, accurate and timely imaging surveillance is critical to monitor disease status, assess response to bridging therapies and ensure that patients remain within transplant criteria throughout the waiting period. All major international guidelines recommend abdominal ultrasound (US), with or without alpha-fetoprotein (AFP) [4–7], as the standard surveillance tool for patients at risk of HCC. However, US carries well-recognized limitations in cirrhotic patients, including poor acoustic windows related to parenchymal changes, increased BMI and hepatic steatosis, resulting in suboptimal sensitivity for HCC detection, particularly at early stages (47–63%) [8–11]. These limitations are especially relevant in the transplant waitlist setting, where missed or delayed diagnosis of tumor progression can lead to patient dropout and loss of transplant opportunity.

Contrast-enhanced MRI, particularly with hepatobiliary-specific contrast agents such as gadoxetate disodium, has demonstrated superior diagnostic performance compared to US, with significantly higher HCC detection rates (86% vs. 28%, *p* < 0.001) and lower false-positive rates (3% vs. 6%, *p* = 0.004) in patients with HCC risk primarily due to HBV infection [8]. Reflecting this evidence, MRI has become the most frequently used imaging modality (> 50%) for HCC surveillance in tertiary transplant centers [12], despite not being recommended by current guidelines due to concerns over acquisition time, costs and availability.

To address these practical barriers, abbreviated MRI protocols (AMRI) have been proposed, combining selected sequences, including T2-weighted imaging, diffusion-weighted imaging (DWI) and dynamic or hepatobiliary phase acquisitions, to reduce acquisition time, costs and motion artifacts while maintaining diagnostic accuracy. Although several studies have validated AMRI for HCC detection in general screening populations [13, 14], no study to date has specifically evaluated its performance in patients on the liver transplant waitlist, a population in which liver-directed therapies—traditionally considered exclusion criteria—are now a standard component of clinical management.

Therefore, the aim of our study was to compare the diagnostic performance of three different AMRI protocols, simulated by extraction from full standard hepatobiliary-contrast MRI examinations, in patients with HCC undergoing imaging surveillance as candidates for liver transplantation.

## Methods

### Study design

We conducted a retrospective cross-sectional study at Policlinico Umberto I-Sapienza University of Rome. Written informed consent was obtained from all participants, ensuring ethical compliance and confidentiality when reviewing historical data. We identified consecutive patients with cirrhosis, eligible for liver transplantation, enrolled in a MRI-based surveillance program, who underwent an abdominal MRI using hepatobiliary-specific contrast agent between January 2024 and December 2024. The inclusion criteria were: liver transplant wait-listing; medical history of cirrhosis; unequivocal liver cirrhosis morphology on prior imaging records. Patients who already had HCC at the time of enrollment were included in case of single ≤ 5 cm in maximum diameter or up to 3 solitary lesions smaller than 3 cm [15]. Patients who previously had liver-directed therapy (e.g., surgical resection, MWA, TACE, RFA, SBRT) were included only if the immediate prior MRI demonstrated no evidence of HCC [16]. The exclusion criteria were: known malignancy other than HCC; severe motion imaging artifacts affecting the diagnostic interpretability; intravenous contrast administration failure; no follow-up imaging or procedures. Patients’ characteristics available from medical records comprised: age, sex and etiology of underlying liver disease. Diagnosis of HCC was then confirmed by histopathological assessment and/or imaging follow-up within a 12-month period, ultimately determining the final HCC status.

### Image acquisition and image analysis

Each patient fulfilling inclusion criteria was evaluated with a gadoxetic acid- (Primovist; Bayer Healthcare) or gadobenic acid- (MultiHance, Bracco) enhanced complete MRI protocol (cMRI) using the 3 T system (Magnetom Vida; Siemens Healthcare). The choice of either contrast medium was based on: patient-specific contraindications; known hypersensitivities reaction and contrast agent availability, while also taking into account the absence of statistically significant difference in their diagnostic performance [17]. The routine cMRI included: coronal T2-weighted single-shot turbo spin-echo; axial T2 single-shot turbo spin-echo; axial T2 single-shot turbo spin-echo with fat saturation; diffusion-weighted imaging (DWI) with three B value (50, 400; 800 mm^3^); pre-contrast axial T1-weighted in and out of phase; pre-contrast axial T1-weighted spoiled gradient echo; dynamic axial T1-weighted spoiled gradient echo fat suppressed sequences performed during the pre-contrast, arterial, portal venous and equilibrium phase according to LI-RADS technical recommendations [18]; hepatobiliary phase (HBP) axial T1-weighted fat suppressed. The patients received a standard dose (0.025 mmol per kg of body weight of Gadoxetate disodium; Primovist; Bayer Healthcare or 0.1 mmol per kg of body weight of gadobenic acid; MultiHance; Bracco).

To simulate three abbreviated MRI protocols (non-enhanced abbreviated MRI (NE-AMRI); dynamic contrast-enhanced abbreviated MRI (Dyn-AMRI) and hepatobiliary phase abbreviated MRI (HBP-AMRI)), a set of sequences were extracted from cMRI. The NE-AMRI included axial T2-weighted fat suppressed sequence; DWI sequences; axial T1-weighted spoiled gradient echo sequence. The Dyn-AMRI included: axial T2-weighted with fat saturation sequence; DWI sequences; axial T1-weighted multiphase sequences (non-enhanced, arterial phase, portal venous phase and delayed phase). The HBP-AMRI included: axial T2-weighted with fat saturation sequence; DWI sequences; HBP axial T1-weighted fat suppressed sequence. Three abdominal radiologists with at least three years of experience in HCC screening independently interpreted the three sets of reading protocols following randomization, blinded to all prior imaging data and without the possibility to view any other image acquired as part of complete MRI protocol. A washout period of at least three weeks was established between reviews of the different abbreviated protocols of the same patient to avoid bias. On average, the acquisition time was above 20 min for cMRI, approximately 17 min for Dyn-AMRI, 15 min for NE-AMRI and HBP-AMRI (Table 1).

**Fig. 1:**
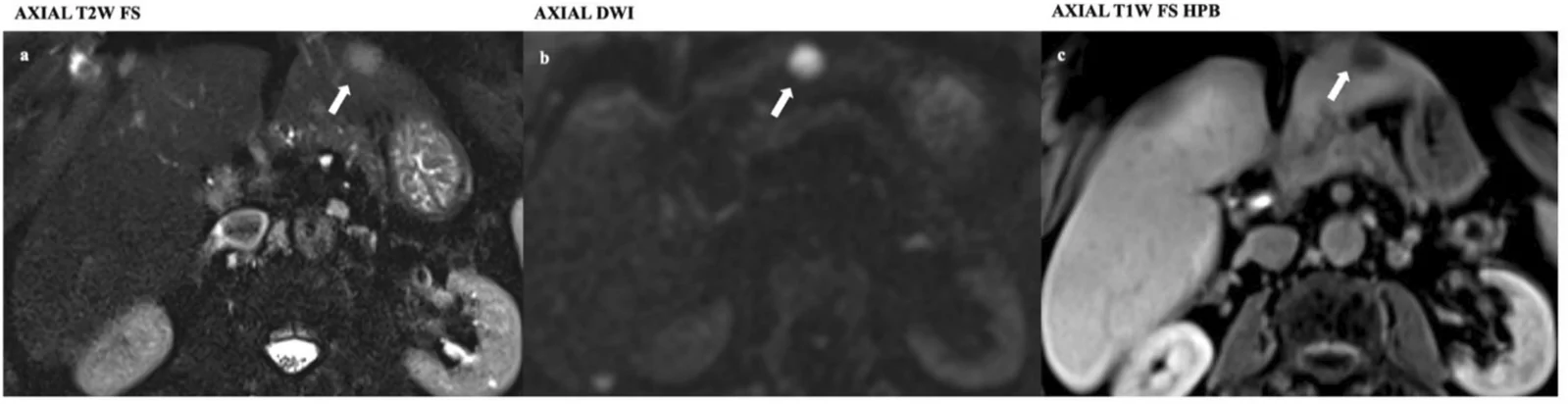
Example of HCC nodule in segment III identified with HPB-AMRI. On axial T2-weighted with fat saturation image (T2W FS), the nodule appears hyperintense; on axial DWI image, the nodule demonstrates restricted diffusion, and on axial T1-weighted with fat saturation image acquired on hepatobiliary phase (T1W FS HPB), the nodule appears markedly hypointense

**Table 1:** cMRI, Dyn-AMRI, HBP-AMRI and NE-AMRI protocols with corresponding average acquisition times (min:s)

Sequence	Acquisition time (AT)
cMRI	
Localizer	00:21
Coronal T2 HASTE	00:52
Axial T2 HASTE	01:02
Axial T2 HASTE FS	01:15
Axial DWI	03:09
Axial T1 DIXON in-opp	00:15
Axial T1 VIBE FS pre-contrast	00:14
Care bolus/timing	00:20
Axial multiphase T1 VIBE FS AP	00:17
Delay	00:38
Axial multiphase T1 VIBE FS PVP	00:14
Delay	00:42
Axial multiphase T1 VIBE FS TP	00:14
Coronal multiphase T1 VIBE TP	00:15
Axial T1 VIBE FS HPB	00:12
Coronal T1 VIBE HPB	00:13
Set up time	10:00
Total AT cMRI	20:20
NE-AMRI	
Localizer	00:21
Axial T2 HASTE FS	01:15
Axial DWI	03:09
Axial T1 VIBE DIXON in-opp	00:15
Set up time	10:00
Total AT NE-AMRI	15:00
Dyn-AMRI	
Axial T2 HASTE FS	01:15
Axial DWI	03:09
Axial multiphase T1 pre-contrast	00:14
Care bolus/timing	00:20
Axial multiphase T1 VIBE FS AP	00:17
Delay	00:38
Axial multiphase T1 VIBE FS PVP	00:14
Delay	00:42
Axial multiphase T1 VIBE FS EP	00:14
Set up time	10:00
Total AT cMRI	1705
HBP-AMRI	
Localizer	00:21
Axial T2 HASTE FS	01:15
Axial DWI	03:09
Axial T1 FS HPB	00:12
Set up time	10:00
Total AT HBP-AMRI	14:90

AP: arterial phase; DWI: diffusion-weighted imaging; EP: equilibrium phase; FS: fat saturated; HASTE: half-Fourier acquisition single-shot turbo spin-echo imaging; in-opp: in–out phase; HBP: hepatobiliary phase; PVP: portal venous phase

### Reference standard

Each patient was followed from the moment of first cMRI exam until HCC status could be determined within 12 months. The final HCC status (positive or negative) was determined according to a composite reference standard: if pathological examination identified one HCC; if a follow-up multiphase CT or MRI imaging identified one LI-RADS category 4 (probably HCC) or 5 (definitely HCC); if a liver-directed treatment was done on the basis of complete MRI or CT report [19, 20].

### Statistical analysis

Both ordinal and binary evaluation systems were evaluated for intra- and inter-reader agreement on a per-patient basis. The intra-reader agreement involved comparing the outcomes of Dyn-AMRI, HBP-AMRI, NE-AMRI and cMRI in pairs across all operators, while the inter-reader agreement assessed the scoring reproducibility of the different imaging protocol. Fleiss’ and Cohen’s kappa statistics were calculated, with a 95% confidence intervals (CI) as appropriate. Imaging tests determining a binary HCC screening outcome were evaluated in sets of two for interchangeability and equivalence, following statistical methodologies based on generalized estimating equations (GEE) [21–23]. All statistical analyses were conducted using SPSS, version 26 (IBM).

**Fig. 2:**
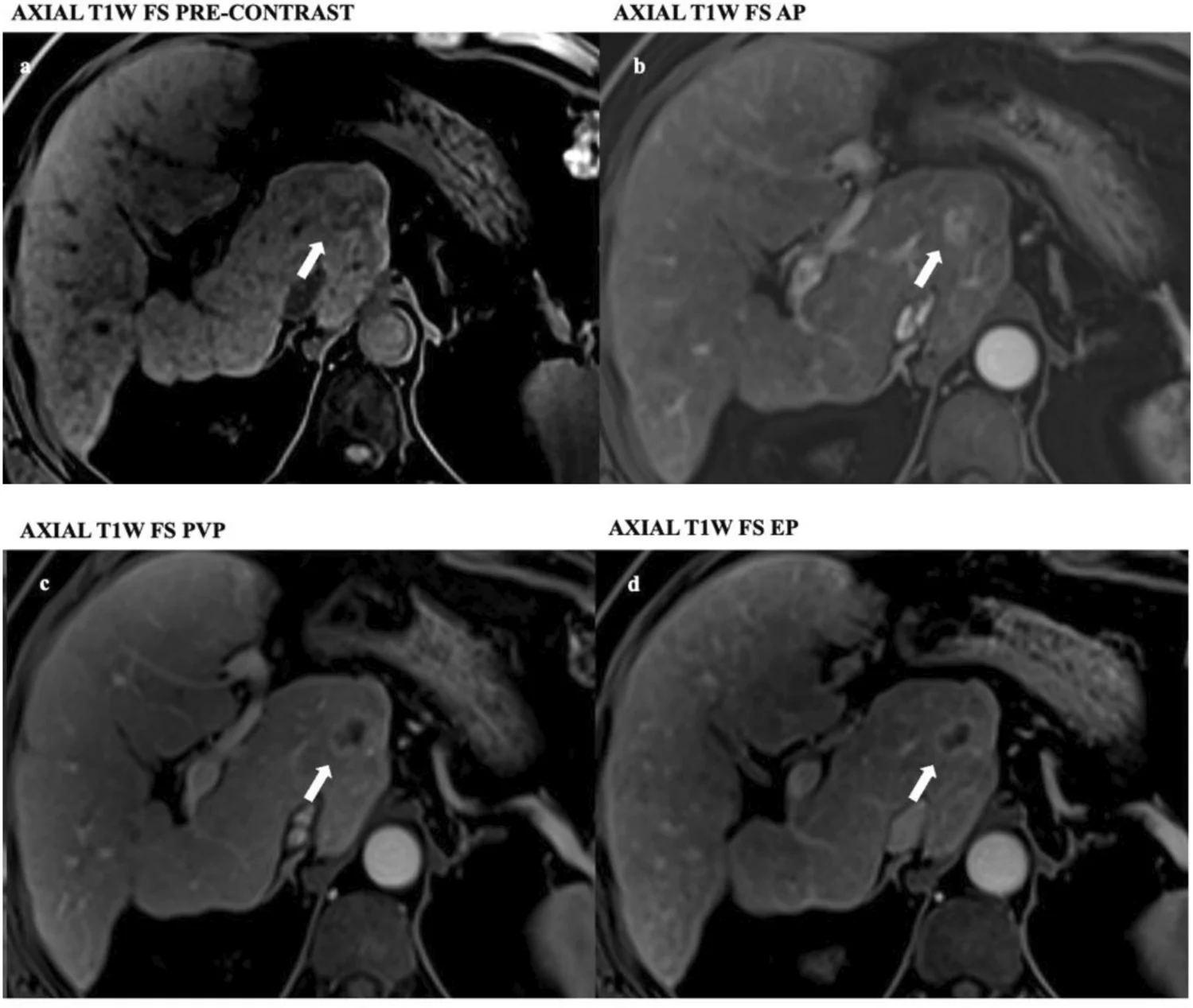
Example of HCC nodule in segment I identified with Dyn-AMRI. On axial T1-weighted with fat saturation image acquired before and after contrast medium administration in various phases, the nodule has the following features: on pre-contrast image is hypointense; on arterial phase image (AP) is markedly hyperintense and on portal venous phase image (PVP) and on equilibrium phase (EP) is hypointense with enhancing capsule

## Results

### Patient population

Two hundred and fifteen consecutive patients fulfilling the inclusion criteria underwent a standard liver MRI with hepatobiliary-specific contrast agent (Table 2). A total 68/195 (34.9%) were administered with Primovist, the remainder with MultiHance (127/195; 65.1%). Fifteen patients were excluded because of severe motion artifacts and five patients were excluded due to inability to determine the final HCC status at 12 months. Thus, 195 patients were included in the statistical analysis. As determined by the composite reference standard, the prevalence of active HCC was 61/195 patients (31.3%). The final HCC status was determined using histopathological criteria in 20/61 cases following transplantation; the remainder were assessed with radiological criteria (Figs.  1 and 2).

**Table 2:** Patient population demographics and clinical characteristics

Frequency (%)		
Sex	M	136 (69.0%)
Etiology of liver disease	Viral	72 (36.5%)
	Other etiologies	63 (32.3%)
	ALD	40 (20.3%)
	MASLD	14 (7,2%)
	PSC Autoimmune	5 (2.5%) 1 (0.5%)
HCC treatment performed before imaging follow-up		105 (53.3%)
	RFTA	44 (41.9%)
	Surgical resection	23 (21.9%)
	TACE	22 (20.9%)
	MWA	14 (13.3%)
	SIRT	2 (1.9%)
LI-RADS	LR < = 3	134 (68.7%)
	LR 4	21 (10.7%)
	LR 5	40 (20.5%)
Average (SD)		
Age		63.8 (9.0)

ALD: alcohol-associated liver disease; MASLD: metabolic dysfunction-associated liver disease; MWA: microwave ablation; PSC: primary sclerosing cholangitis; RFTA: radiofrequency thermoablation; SIRT: selective internal radiation therapy; TACE: transarterial chemoembolization

**Table 3:** Equivalence analysis (per-reader and per-average protocol). Per-reader HCC screening performance for the three readers and an estimated average reader

Reader	Protocol	Sensitivity (95%CI)	Specificity (95%CI)	Accuracy (95% CI)
Reader 1	cMRI	0.96[0.92–0.99]	0.93[0.90–0.97]	0.95[0.91–0.99]
	HPB-AMRI	0.94[0.89–1.00]	0.84[0.78–0.90]	0.90[0.85–0.96]
	Dyn-AMRI	0.88 [0.84–0.92]	0.81[0.76–0.86]	0.86 [0.81–0.90]
	NE-AMRI	0.89[0.82–0.95]	0.90[0.96–0.84]	0.89[0.83–0.95]
Reader 2	cMRI	0.95[0.97–0.99]	0.93[0.89–0.97]	0.95[0.91–0.98]
	HPB-AMRI	0.93[0.88–0.99]	0.86[0.81–0.92]	0.90[0.85–0.96]
	Dyn-AMRI	0.96 [0.93–0.98]	0.95 [0.92–0.98]	0.96 [0.93–0.98]
	NE-AMRI	0.90[0.84–0.95]	0.91[0.85–0.97]	0.90[0.84–0.96]
Reader 3	cMRI	0.98 [0.93–1]	0.94[0.99–0.90]	0.96[0.91–1]
	HPB-AMRI	0.93[0.87–0.98]	0.85[0.79–0.91]	0.90[0.84–0.96]
	Dyn-AMRI	0.95 [0.93–0.98]	0.91 [0.88–0.95]	0.94 [0.91–0.97]
	NE-AMRI	0.90[0.85–0.96]	0.90[0.84–0.96]	0.90[0.84–0.96]
Average	cMRI	0.95[0.92–0.99]	0.99[0.95–1]	0.97[0.93–1]
	HPB-AMRI	0.93[0.88–0.98]	0.94[0.88–0.99]	0.93[0.88–0.99]
	Dyn-AMRI	0.94 [0.90–0.97]	0.96 [0.93–0.98]	0.94 [0.91–0.97]
	NE-AMRI	0.90[0.84–0.96]	0.90[0.84–0.96]	0.90[0.84–0.97]
Average differences	cMRI- HBP-AMRI	0.03[− 0.02–0.09]	0.06 [0.00–0.11]	0.06[0.01–0.12]
	cMRI- Dyn-AMRI	0.02 [0.02- 0.02]	0.03 [0.03-0.02]	0.03 [0.03− 0.03]
	cMRI-NE- AMRI	0.07[0.01–0.13]	0.10[0.04–0.16]	0.10[0.04–0.16]
	HPB-AMRI- Dyn-AMRI	− 0.00 [− 0.01–0.00]	− 0.02 [-0.04- 0.01]	− 0.01 [-0.02–0.00]
	HPB-AMRI- NE-AMRI	0.04[− 0.00–0.02]	0.04[0.00–0.08]	0.04[0–0.08]
	Dyn-AMRI- NE-AMRI	0.03 [0.02—0.05]	0.06 [0.04–0.08]	0.04 [0.03–0.06]

c-AMRI: complete abbreviated MRI; Dyn-AMRI: dynamic abbreviated MRI; HPB-AMRI: hepatobiliary phase abbreviated MRI; NE-AMRI: non-enhanced abbreviated MRI

### Equivalence analysis

The average reader sensitivity, specificity and accuracy were 95%, 99% [95%CI 0.95–1] and 97% [95%CI 0.933–1] for cMRI, 94% [95%CI 0.905–0.968], 96% [95%CI 0.935–0.985] and 94% [95%CI 0.914–0.974] for Dyn-AMRI, 93% [95%CI 0.876–0.988], 94% [95%CI 0.88–0.993] and 93% [95%CI 0.876–0.991] for HBP-AMRI, 90% [95%CI 0.847–0.959], 90% [95%CI 0.838–0.964] and 90% [95%CI 0.839–0.966] for NE-AMRI. No significant average difference was found between Dyn-AMRI and HBP-AMRI. Among all the comparisons, the highest average difference was found between cMRI and NE-AMRI. No statistically significant differences in specificity, sensitivity and accuracy were found when applying all three abbreviated protocols in the evaluation of patients previously undergoing liver-directed therapy (Table 3).

**Table 4:** Diagnostic reliability: treated versus non-treated

	Average Between-Group difference [95% CI]	p
InterChangeability_Dyn-AMRI	− 0.01 [− 0.03–0.02]	0.529
InterChangeability_HBP-AMRI	− 0.00 [− 0.03–0.02]	0.819
InterChangeability_NE-AMRI	− 0.01 [− 0.04–0.01]	0.351
InterChangeability_T2fatsat	− 0.01 [− 0.04–0.01]	0.309
InterChangeability_DWI	− 0.02 [− 0.05–0.01]	0.263
InterChangeability_HBp	− 0.01 [− 0.04–0.01]	0.400
Agreement_TOT_Dyn-AMRI	0.03 [− 0.02–0.08]	0.258
Agreement_TOT_HBP-AMRI	0.01 [− 0.05–0.08]	0.662
Agreement_TOT_NE-AMRI	0.02 [− 0.04–0.09]	0.482
Agreement_Dyn-AMRI_HPB-AMRI	− 0.02 [− 0.08–0.04]	0.597
Agreement_Dyn-AMRI_NE-AMRI	− 0.02 [− 0.07–0.06]	0.832
Agreement_HPB-AMRI_NE-AMRI	0.01 [− 0.06–0.08]	0.796
Agreement_TOT_DWI	0.06 [− 0.01–0.13]	0.087
Agreement_TOT_T2	0.04 [− 0.02–0.10]	0.159
Agreement_TOT_HBp	− 0.01 [− 0.07–0.06]	0.808
Average difference_TOT_Dyn-AMRI	− 0.01 [− 0.06–0.03]	0.548
Average difference_TOT_HPB-AMRI	− 0.02 [− 0.07–0.03]	0.380
Average difference_TOT_NE-AMRI	− 0.04 [− 0.09–0.02]	0.232
Average difference_Dyn-AMRI_HPB-MRI	− 0.01 [− 0.07–0.05]	0.751
Average difference_Dyn-AMRI_NE-AMRI	− 0.02 [− 0.09—0.04]	0.490
Average difference_HBP-AMRI-NE-AMRI	− 0.01 [− 0.07–0.05]	0.669

c-AMRI: complete abbreviated MRI; Dyn-AMRI: dynamic abbreviated MRI; HBP: hepatobiliary phase; HPB-AMRI: hepatobiliary phase abbreviated MRI; NE-AMRI: non-enhanced abbreviated MRI

### Agreement and interchangeability analysis

For per-patient HCC detection, the intra-reader kappa statistics [95% CI] between cMRI and the three abbreviated AMRI proposed were 0.89, 0.87 and 0.83 for HBP-AMRI, Dyn-AMRI and NE-AMRI, respectively. The intra-reader-agreement probability between the three proposed AMRI protocols and the cMRI was greater than 90% in all considered combinations with a value near perfect at 0.99 for cMRI and HBP-AMRI and for cMRI and Dyn-AMRI. The interchangeability analysis revealed no significant difference between HBP-AMRI and cMRI and between cMRI and Dyn-AMRI, and a value only slightly in favor of cMRI in the case of comparison between cMRI and NE-AMRI. No statistically significant differences were found regarding the agreement and interchangeability analysis in the application of all three abbreviated protocols to patients previously undergoing liver-directed therapy (Tables 4 and 5).

**Table 5:** Agreement and interchangeability analysis (per-average protocol and per sequence)

	cMRI vs HBP-AMRI	cMRI vs Dyn-AMRI	cMRI vs NE-AMRI	HPB-AMRI vs Dyn-AMRI	HBP-AMRI vs NE-AMRI	Dyn-AMRI vs NE-AMRI
Cohen’s k	0.89 [0.83–0.96]	0.87 [0.81–0.94]	0.83 [0.75–0.90]	0.79 [0.71–0.87]	0.79 [0.69–0.86]	0.81 [0.73–0.89]
Agreement %	0.99 [0.99–1.00]	0.99 [0.98–1.03]	0.95 [0.94–0.96]	0.98 [1.02–0.95]	0.94 [0.94–0.94]	0.97 [0.94–1.01]
Interchangeability	− 0.02 [− 0.04–0.00]	0.01 [− 0.01–0.03]	0.03 [0.00–0.06]	− 0.03 [− 0.06—0.02]	0.05 [0.02–0.08]	0.03 [− 0.01–0.06]

c-AMRI: complete abbreviated MRI; Dyn-AMRI: dynamic abbreviated MRI; HPB-AMRI: hepatobiliary phase abbreviated MRI; NE-AMRI: non-enhanced abbreviated MRI

### Inter-reader-agreement correlation

The highest inter-reader-agreement value of 96% was found in cMRI, followed by Dyn-AMRI with a value of 89% and HBP-AMRI, with a value of 88%. Dynamic sequences and HBP sequence showed the highest kappa statistics with the final diagnosis with values of 0.87 and 0.84 [95%CI 0.911-0.762], respectively. The lowest value of 0.76 was reported for DWI sequence (Table 6 and 7).

**Table 6:** Inter-reader-agreement correlation

	Inter-reader-agreement correlation
cMRI	0.96 [0.95–0.97]
HBP-AMRI	0.88 [0.83–0.92]
Dyn-AMRI	0.89 [0.86–0.91]
NE-AMRI	0.75 [0.69–0.80]
DWI sequence	0.95 [0.93–0.60]
HBP sequence	0.96 [0.96–0.97]
T2-weighted sequence	0.96 [0.94–0.96]
Dynamic sequences	0.89 [0.86–0.91]

c-AMRI: complete abbreviated MRI; Dyn-AMRI: dynamic abbreviated MRI; HBP: hepatobiliary phase; HPB-AMRI: hepatobiliary phase abbreviated MRI; NE-AMRI: non-enhanced abbreviated MRI

**Table 7:** Per sequence Kappa with final cMRI

		Cohen’s K
	T2w sequence	0.83 [0.76–0.91]
cMRI vs	HBP sequence	0.84 [0.76–0.91]
	Dynamic sequences	0.87 [0.80–0.92]
	DWI sequence	0.76 [0.68–0.85]

cMRI: complete MRI; HBP: hepatobiliary phase

## Discussion

We compared the diagnostic performance of three sets of AMRI extracted from a complete contrast-enhanced MRI protocol. Our results confirmed the previous literature data, showing high per-patient level detection sensitivity and specificity for all the three AMRI protocols proposed [14, 24, 25], without significant difference between HBP-AMRI and cMRI and between Dyn-AMRI and cMRI; however, only a significant slight average difference was found between NE-AMRI and cMRI. The results obtained from the equivalence analysis, in addition to confirming what has already been demonstrated about Dyn-AMRI, showed that HBP-AMRI is equivalent to cMRI and Dyn-AMRI, despite the lack of ancillary and contrast features that would allow a nodule to be classified according to LI-RADS criteria. Moreover, our study revealed a near perfect level of agreement between HBP-AMRI and cMRI and between Dyn-AMRI and cMRI, respectively, of 99.8% and 99.9%, showing their interchangeability in HCC detection.

The results about NE-AMRI, the simplest among the three AMRI, regarding sensitivity and specificity, were in the higher range for specificity than those previously demonstrated [26, 27], underlining its potential role, especially in case of periodic repetitive examinations.

We also evaluated the inter-reader-agreement correlation, data that are lacking from the literature, with high values for all the three abbreviated protocols, especially for HBP-AMRI, revealing its repeatability even with different levels of experience.

To our knowledge, almost all the studies in literature consider previous directed-liver treatments, often critical in downstaging of patients eligible to liver transplant, among the main exclusion criteria. In our study, no statistically significant differences were found in this subgroup population regarding diagnostic performance of all the three AMRI described. Moreover, no significant differences were found regarding the diagnostic performance of single MRI sequences in detecting HCC in patients non-treated and treated. This result, although in need of further studies on larger samples, opens the possibility to the use of the abbreviated protocol also in secondary surveillance of already treated patients (28).

Our study had several limitations. First of all, the retrospective study design, with the consequent extraction of Dyn-AMRI from a cMRI with hepatobiliary-specific contrast agent, results in the inability to evaluate a dynamic protocol with extracellular contrast agent. Another important limitation of our study was the need to use a composite reference standard, due to the lack of histopathological correlation for all patients. However, a large subgroup of patients has undergone direct liver therapy presenting a LI-RADS of 4 or 5, as in use in clinical practice even in the absence of histological confirmation.

In summary, all three abbreviated protocols showed high levels of sensitivity and specificity for HCC detection in cirrhotic patients. In addition, this study demonstrated the equivalence and the interchangeability of cMRI and both Dyn-AMRI and HBP-AMRI and only a slight difference in favor of the full protocol compared with NE-AMRI. Thus, both HBP-AMRI and Dyn-AMRI could be considered equivalent to cMRI in surveillance of cirrhotic patients. Even more relevant than literature data is that no statistically differences were found in the application of AMRI in patients treated.

## Abbreviations

AFP: Alpha-fetoprotein
ALD: Alcohol-associated liver disease
AMRI: Abbreviated magnetic resonance imaging
AP: Arterial phase
AT: Acquisition time
BCa: Bias-corrected and accelerated bootstrap
BOPTA: Gadobenate dimeglumine (Gd-BOPTA)
CI: Confidence interval
cMRI: Complete magnetic resonance imaging
DWI: Diffusion-weighted imaging
Dyn-AMRI: Dynamic contrast-enhanced abbreviated MRI
EOB: Gadoxetate disodium (Gd-EOB-DTPA)
EP: Equilibrium phase
FS: Fat saturated
GEE: Generalized estimating equations
HASTE: Half-Fourier acquisition single-shot turbo spin-echo
HBP: Hepatobiliary phase;
HBP-AMRI: Hepatobiliary phase abbreviated MRI
HCC: Hepatocellular carcinoma
LI-RADS: Liver imaging reporting and data system
MASLD: Metabolic dysfunction-associated steatotic liver disease
MAFLD: Metabolic dysfunction-associated fatty liver disease
MRI: Magnetic resonance imaging
MWA: Microwave ablation
NE-AMRI: Non-enhanced abbreviated MRI
PVP: Portal venous phase
PSC: Primary sclerosing cholangitis
RFA: Radiofrequency ablation
RFTA: Radiofrequency thermoablation
SBRT: Stereotactic body radiation therapy
SD: Standard deviation
SIRT: Selective internal radiation therapy
TACE: Transarterial chemoembolization
T1W FS: T1-weighted fat-saturated sequence
T2W FS: T2-weighted fat-saturated sequence
TOT: Total agreement
TP: Transitional phase
US: Ultrasound
VIBE: Volumetric interpolated breath-hold examination

## Declarations

### Human or animals rights

This study was conducted in accordance with ethical standards, involving human participants.
